# Management algorithm for failed gastric pull up reconstruction of laryngopharyngectomy defects: case report and review of the literature

**DOI:** 10.1186/s40463-016-0153-3

**Published:** 2016-07-22

**Authors:** Oleksandr Butskiy, Donald W. Anderson, Eitan Prisman

**Affiliations:** Division of Otolaryngology – Head and Neck Surgery, Department of Surgery, Vancouver General Hospital & University of British Columbia, Vancouver, BC Canada; Gordon & Leslie Diamond Health Care Centre, 4th. Fl. 4299B-2775 Laurel Street, Vancouver, BC V5Z 1M9 Canada

**Keywords:** Pharyngoesophagectomy, Gastric pull up, Anterolateral thigh, Head and neck cancer, Head and neck reconstruction

## Abstract

**Background:**

Gastric pull up remains a popular reconstructive option for pharyngoesophagectomy defects extending to thoracic inlet. Gastric necrosis is a dreaded complication of gastric pull up reconstruction and few studies report on management of this complication.

MEDLINE, EMBASE, and Web of Science™ databases were searched for publications in the last 25 years on gastric pull up reconstruction following pharyngoesophagectomy. The rates of complications related to gastropharyngeal anastomosis were extracted, and methods of managing gastric necrosis were noted.

Forty seven case series were identified reporting on the use of gastric pull up for reconstruction of pharyngoesophageal defects. Mortality rate varied from 0 to 33 % with a weighted average of 8.6 %. In 39 % of patients, mortality was either caused or directly related to failure of the gastropharyngeal anastomosis. The reported rate of gastric necrosis ranged from 0 to 24 % resulting in a 28 % mortality. Options for managing gastric necrosis included: temporary cervical diversion, free jejunum flap, colonic interposition, tubed radial forearm flap, deltopectoralis and pectoralis myocutaneous flaps.

**Case presentation:**

We present the first case of an anterolateral thigh flap rescue of gastric necrosis after gastric pull up reconstruction. The case report is followed by a review of literature on management of gastric pull up failures.

**Conclusion:**

Based on the extracted information, we propose an algorithm for managing gastric pull up failure following pharyngoesophageal reconstruction.

## Background

Reconstructing circumferential pharyngoesophagectomy defects remains a challenging procedure for reconstructive surgeons. Despite a multitude of vascularized free tissue transfers options popularized in the 1980s and 1990s [1, 2], Gastric pull up (GPU) remains a popular choice for pharyngoesophageal reconstruction. The robust blood supply offered by the gastric mucosa, requirement for only one mucosal anastomosis, and lack of microvascular anastomosis are noted advantages of the GPU. Despite these advantages, a rare but critical complication is proximal necrosis of the GPU leading to dehiscence at the gastropharyngeal anastomosis. If not managed properly, the dehiscence will result in mediastinitis, sepsis, and death. Thus, all reconstructive surgeons offering GPU reconstructions should be familiar with the surgical management of this dreaded complication. Unfortunately, the literature on this topic is scant [3]. To the best of our knowledge, we present the first case report of an anterolateral thigh free flap (ALT) rescue of a failed GPU pharyngoesophageal reconstruction. A review of the available literature and a management algorithm of gastro-pharyngeal anastomotic failure following GPU pharyngoesophageal reconstruction are presented.

## Case presentation

A 69-year-old male presented to the otolaryngology office with complaints of right sided neck mass and otalgia. His past medical history was significant for 50 years of smoking, regular alcohol use, and colonic adenocarcinoma managed with a colectomy several years prior. He was diagnosed with T4aN2aM0 hypopharyngeal carcinoma involving the right pyriform sinus with a single 4 cm metastasis to the right level V. He was offered surgical resection followed by GPU reconstruction and planned adjuvant radiotherapy.

Of note, during surgical planning, it was felt that the mediastinal esophagus was likely not involved with the tumor. Thus, tumor resection was expected to produce a circumferential pharyngeal defect extending into the cervical esophagus, but not the mediastinal esophagus. Faced with such a defect, to avoid the morbidity associated with entering the abdominal cavity, many surgeons would advocate for reconstruction with a tubed cutaneous free flap rather than with the GPU [1]. However, at our institution one of the authors (D.W.A) working alongside the thoracic surgery team has been able to achieve better functional outcomes with the use of GPU as compared to reconstruction with tubed cutaneous free flaps. After careful consultation with the thoracic surgery team, a joint decision was made to pursue GPU reconstruction.

A laryngopharyngectomy and right modified radical neck dissection were performed without complication. Following the resection, the thoracic surgery team proceeded with the esophagectomy and gastric mobilization. Gastric mobilization was hindered by intrabdominal adhesions related to the previous colectomy as well as dilated gastric veins related to apparent liver cirrhosis. Nevertheless, a well-vascularized and tensionless gastropharyngeal anastomosis was attained and a jejunostomy tube inserted.

Postoperatively, the patient was managed in the intensive care unit due to difficulty weaning from the ventilator. His early postoperative course was complicated by sepsis, and an anastomotic leak was considered despite serosangouinous neck drains and no wound breakdown. He was managed conservatively with antibiotics until postoperative day 7, when he lost vacuum on the negative pressure suction drain in the neck. Dehiscence was confirmed using a water-based dye.

**Fig. 1:**
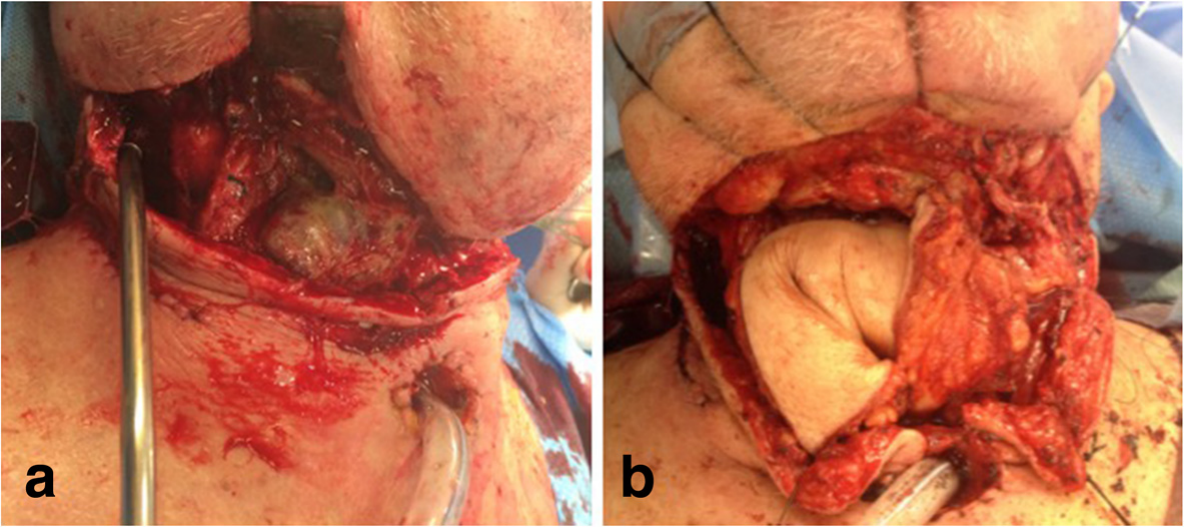
**a** Circumferential necrosis of the stomach at the gastropharyngeal anastomosis. **b** Anterolatral thigh flap folded in a conical design

The patient was then taken to the operating room and found to have circumferential necrosis of the proximal GPU extending inferiorly into the upper mediastinum (Fig. 1a). The necrosis was debrided until well-vascularized gastric mucosa was reached. A large defect remained extending from the distal oropharynx to the proximal superior mediastinum. The reconstructive options to re-establish the continuity of the alimentary tract in this patient were severely limited. Due to the patient’s history of colonic resection and recent gastric pull up, intra abdominal tissue transfer, such as jejunal transfer or colonic interposition were not available. The two remaining options included a vascularized free tissue transfer or creating a controlled pharyngeal fistula and over sewing the proximal stomach. A 20 cm by 15 cm elliptical ALT flap was chosen as the donor free tissue transfer, and was folded on itself in conical design to reconstruct a neopharynx (Fig. 1b).

**Fig. 2:**
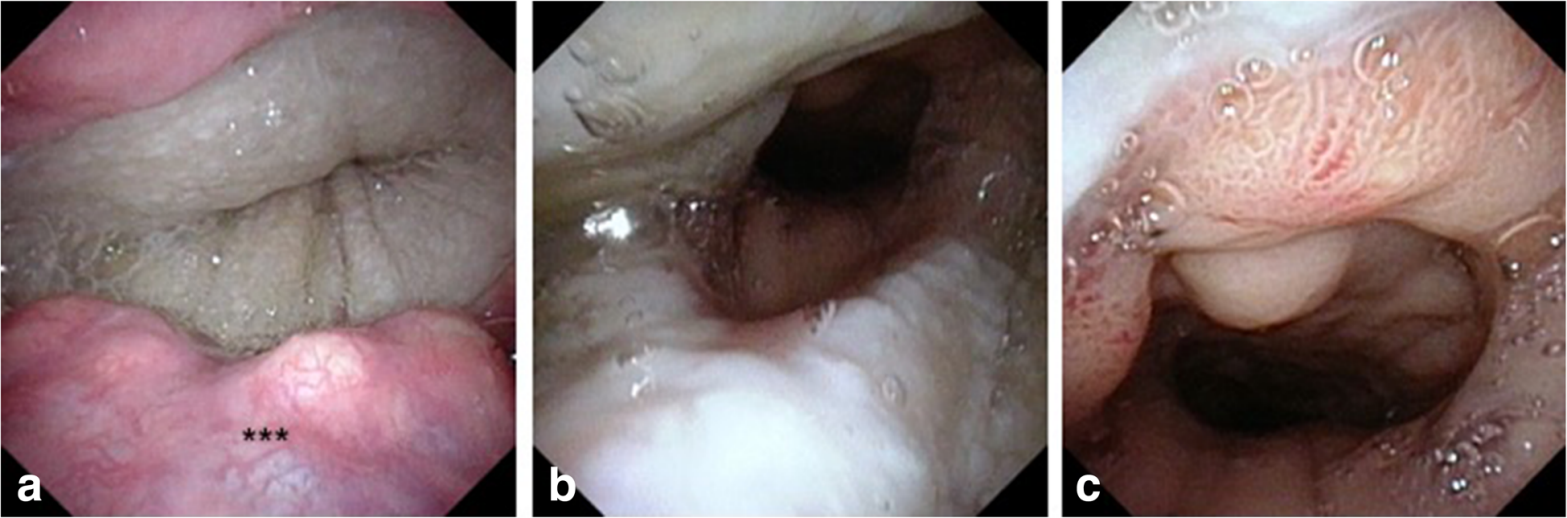
Endoscopic view of anastomosis one month following anterolateral thigh rescue of gastric pull up failure. **a** Pharyngo-cutaneous anastomosis. **b** cutaneo-gastric anastomosis. **c** gastric mucosa distant to the anterolateral thigh flap. ***base of the tongue

**Fig. 3:**
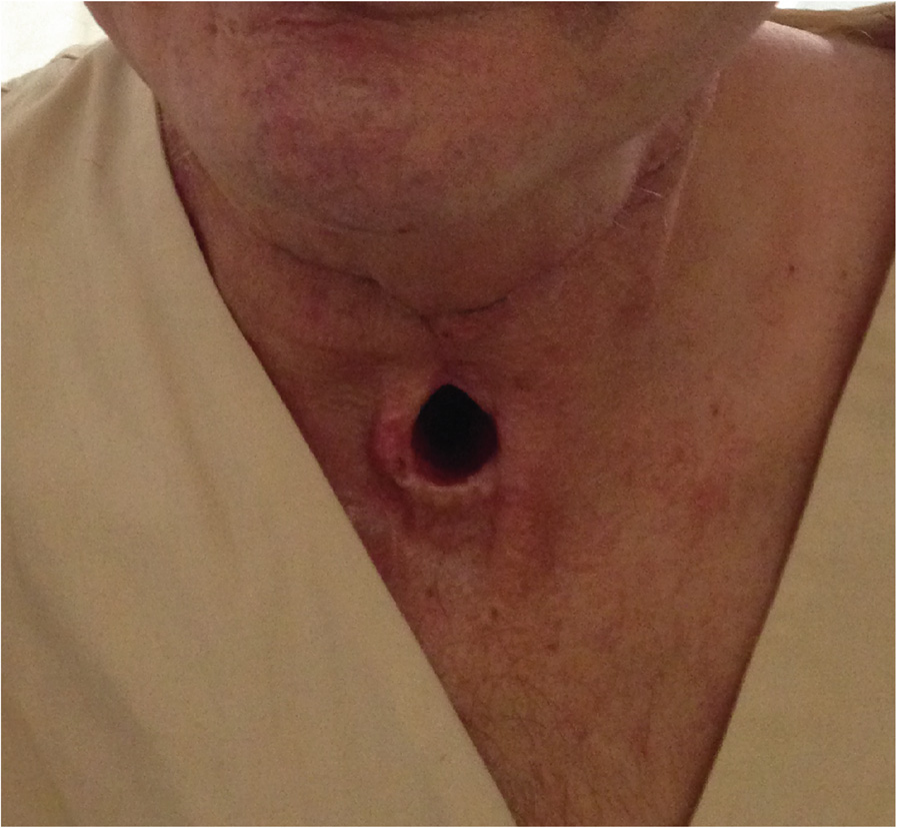
Patient’s laryngostoma three months after the operation

Postoperatively, the patient spent 22 days in the intensive care unit and another month in the hospital undergoing rehabilitation and addressing psychosocial issues. His jejunostomy tube was removed prior to discharge as he was supporting himself nutritionally with a pureed diet. An endoscopic view of the ALT anastomosis one month post reconstructive surgery is shown in Fig. 2. At four months recovery he remains on an oral diet. His laryngostoma is shown in Fig. 3.

## Literature review

We searched MEDLINE, EMBASE, and Web of Science™ databases for English language case reports and case series of GPU reconstruction following pharyngoesophagectomy published from 1990 to 2014. From these studies we extracted the rates of complications related to gastropharyngeal anastomotic failure (fistula, anastomotic leak, gastric necrosis, and anastomotic stricture) and the rates and causes of in-hospital mortality (Table 1). In addition, we noted how authors managed gastric necrosis (Table 2).

**Table 1:** Mortality and gastropharyngeal anastomosis complications after pharyngo-esophagectomy and gastric pull up

Author year	Patients (N)	Anastomotic leak	Necrosis (%)	Anastomotic stricture (%)	In-hospital mortality (%)	Cause of mortality (N)
Mansour [4] -1990	6	1 (17 %)	0	0	0	–
El-Naqeeb [5] -1990	24	1 (4 %)	–	–	0	–
Mehta [30] -1990	75	10 (13 %)	–	–	7 (9 %)	Pulmonary sepsis and respiratory failure (2); PE(1); carotid castrophe(1); MI (2); cirrhosis, ascites, septicemia (1)
Spiro [31] -1991	120	15 (13 %)	5 (4 %)	–	13 (11 %)	Anastomotic leakage, tracheal injury, major arterial bleeding (8); respiratory insufficiency (2); liver failure with sepsis (1); peritonitis after acute pseudomembranous colitis (1); multisystem failure with massive intrapleural bleeding after central venous line injury (1)
Madsen [6] -1992	3	–	–	–	0	–
Carlson [17] -1992	23	6 (26 %)	0	3 (13 %)	2 (9 %)	Ruptured innominate artery after fistula formation (1); MI (1)
Wight [32] -1992	16	3 (19 %)	–	–	2 (13 %)	Cerebrovascular accident and later dehiscence of the anterior part of the pharyngo-gastric anastomosis (1); fistula between trachea and the subclavian artery (1)
Marmuse [33] -1994	20	1 (5 %)	–	–	2 (10 %)	MI (2)
Cahow [34] -1994	59	2 (3 %)	1 (2 %)	4 (7 %)	3 (5 %)	Thoracic duct injury with pneumothorax, MI, heart failure, cardiogenic shock(1); pneumothorax, pneumonic sepsis, disseminated intravascular coagulation, multiple organ failure (1); jejunostomy tube displacement, peritonitis and sepsis (1)
Laterza [35] -1994	49	2 (4 %)	2 (4 %)	–	3 (6 %)	–
Yoshino [7] -1995	4	–	–	–	0	–
Bardini [15] -1995	95	22 (23 %)	10 (11 %)	–	14 (15 %)	Anastomotic leak (5); gastric necrosis (4); other (5)
Shenoy [36] -1996	105	15 (14 %)	10 (10 %)	0	16 (15 %)	Intraoperative death due to injury to the posterior tracheal wall injury (1); pharyngocutaneous fistula (5); obsturctive pulmonary disease, pneumotitis or septicemia (9)
Axon [18] -1997	29	3 (10 %)	0	1 (3 %)	4 (14 %)	–
Azurin [19] -1997	19	1 (5 %)	0	2 (11 %)	1 (5 %)	Intraoperatively discovered cirrhosis, anastomotic leak, acute liver failure, multiorgan failure (1)
Al Ghamdi [37] -1998	15	6 (40 %)	–	2 (13 %)	1 (7 %)	Fistula leading to bronchopneumonia (1)
Wei [38] -1998	69	6 (9 %)	1 (1 %)	–	6 (9 %)	Gastric fundus necrosis (1); chest infection and cardiac problems (2); recurrent tumor (2); cerbrovascular accident (1)
Dudhat [39] -1999	60	5 (8 %)	–	0	5 (8 %)	Pulmonary sepsis (1); MI (2); carotid blow out secondary to anastomotic leak (1); septicaemia related to anastomotic leak (1)
Hartley [40] -1999	41	1 (2 %)	–	–	3 (7 %)	Bronchopneumonia (2); hemorrhage(1)
Sullivan [41] -1999	32	10 (32 %)	–	–	4 (12 %)	Multiorgan failure as a result of uncontrolled neck sepsis due to anastomotic leak and fistula (2); PE (1); MI (1)
Affleck [42] -2000	31	2 (6 %)	–	–	3 (10 %)	–
Martins [43] -2000	30	8 (27 %)	2 (7 %)	–	6 (20 %)	Innominate artery rupture (2); carotid artery rupture (1); pneumonia (1); cardiac arrhythmia (1); pulmpnary embolus (1)
Sagawa [44] -2000	6	1 (17 %)	1 (17 %)	0	1 (17 %)	Gastric necrosis leading to arterial bleeding (1)
Jones [45] -2001	50	1 (2 %)	4 (8 %)	1 (2 %)	–	–
Triboulet [25]-2001	127	20 (16 %)	2 (2 %)	8 (6 %)	–	–
Ullah [46] -2002	26	4 (15 %)	–	5 (19 %)	3 (12 %)	Pneumonia (1); congestive heart failure (1); PE (1)
Wong [8] -2003	12	1 (8 %)	–	–	0	–
Puttawibul [24]-2004	48	4 (8 %)	1 (2 %)	–	1 (2 %)	Fundal necrosis, localized infection and carotid artery blow out(1)
Rossi [9] -2005	4	0	0	0	0	–
Clark [22] -2006	21	10 (48 %)	5 (24 %)	6 (29 %)		–
Llorente Pendas [14] -2006	12	6 (50 %)	–	–	4 (33 %)	Cervical Fistual and Sepsis (2); subphrenic abscess (1); general deterioration and multiple organ failure (1)
Pesko [20] -2006	29	5 (17 %)	0	–	3 (10 %)	Anastomotic leak and systemic sepsis (3)
Daiko [47] -2007	19	2 (11 %)	2 (11 %)	–	2 (11 %)	Necrosis of the stomach (1)
Iseli [10] -2007	7	0	–	0	0	–
Krdžalić [11] -2007	4	1 (25 %)	–	–	0	–
Ferahkose [48] -2008	38	1 (3 %)	2 (5 %)	0	2 (5 %)	Gastric necrsosis with sepsis (2)
Keereweer [3] -2010	19	10 (53 %)	2 (11 %)	–	3 (16 %)	Gastric necrosis and respiratory failure (1); mediastinal hemorrhage (1); carotid blow out (1)
Mansour [12] -2011	5	–	–	–	0	–
Shuangba [16] -2011	208	19 (9 %)	–	7 (3 %)	4 (2 %)	Pneumonitis(1); heart failure(2); hemoperitoneum(1)
Tong [49] -2011	70	4 (6 %)	3 (4 %)	–	3 (4 %)	Pneumonia (3)
Camaioni [50] -2012	23	2 (9 %)	–	–	2 (9 %)	–
Sreehariprasad [51] - 2012	17	1 (6 %)	–	–	0	–
Joshi [52] -2013	32	–	5 (16 %)	–	6 (19 %)	–
Lambert [13] -2013	9	1 (11 %)	–	–	0	–
Sayles [53] -2013	19	9 (47 %)	–	–	–	–
Denewer [21] -2014	32	5 (16 %)	0	3 (9 %)	–	–
Sun [54] -2014	48	4 (8 %)	–	–	–	–

**Table 2:** Rescue of gastric pull up necrosis following pharyngo-esophagectomy

Author year	Patients (N)	Rescue method	Outcome
Bardini [15] -1995	10	8 patients: resection of the necrosis, temporary cervical diversion and delayed reanastomosis; 1 patient: colonic interposition; 1 patient: jejunal free transfer	Four deaths as a result of necrosis
Wei [38] -1998	1	Initially salvaged by controlled pharyngostomy and gastrotomy	Carotid blow out and death
Triboulet [25] -2001	2	Temporary cervical diversion, tubed radial forearm flap	–
		Temporary cervical diversion, deltopectoralis myocutaneous flap	–
Tong [49] -2011	3	Debridement of necrotic stomach and staged reconstruction with pectoralis major myocutaneous flap	Survived

– : no information

Forty-seven studies were identified reporting on a total of 1793 patients who were managed with gastric pull up following pharyngoesophagectomy (Table 1). Mortality rate was reported in 41 studies of 1469 patients. Mortality rate varied from 0 % [4–13] to 33 % [14] with a weighted average of 8.6 % (129 patients). Complications of GPU reconstruction related to pharyngogastric anastomosis were relatively common and varied greatly between the studies. The cause of mortality was reported for 108 patients. In 42 patients (39 %) death was either caused by or was directly related to the failure of gastropharyngeal anastomosis.

The rate of anastomotic leaks was reported to range between 0 % [9] and 23 % [15]. A high index of suspicion for an anastomotic leak is required when faced with increasing edema, erythema, or tenderness of the neck skin flaps that present with a rising white blood cell count. Majority of authors treated asymptomatic and limited leaks with a period of conservative management including nasogastric nutrition and external drainage with variable success. For example, in a retrospective review of 208 patients, Shuangba et al. reported an anastomotic leak rate of 9 % (19 patients). With increased nutritional support and conservative treatment, the anastomotic leak resolved in 15 of these patients. The remainder of the patients had a limited albeit persistent leak that required repair with a pectoralis major rotation flap [16]. Bardini et al. reported on 18 patients treated with conservative measures for limited leaks. 14 patients were treated successfully, but 4 patients died as a result of the anastomotic leaks [15]. Severe leaks were usually treated surgically. For example, Bardini et al. reported on 4 severe leaks, one successfully managed with direct reanastomosis, one with placement of a T tube through the defect to drain saliva and eventual skin flap repair, and two patients were managed by resuturing the posterior wall of the anastomosis while the anterior wall and gastric margins were brought out to the skin [15].

As compared to management of anastomotic leaks, where only a portion of the anastomosis has dehisced, fewer studies report on the management of circumferential gastric necrosis following GPU reconstruction of hypopharngeal defects (Tables 1 and 2). The reported rate of gastric necrosis after GPU reconstruction of hypopharyngeal defects ranged from 0 % [4, 9, 17–21] to 24 % [22] (Table 1). 15 studies reported on both the rate of gastric necrosis and causes of mortality. Out of 40 patients with gastric necrosis in these studies, 11 patients died – a rate of 28 %. Given that many studies were not specific about the cause of death, this mortality rate for gastric necrosis after GPU reconstruction is likely an underestimate. Options for rescuing failed GPU reconstruction included: temporary cervical diversion, free jejunum flap, colonic interposition, tubed radial forearm flap, deltopectoralis and pectoralis myocutaneous flaps (Table 2).

## Discussion

**Fig. 4:**
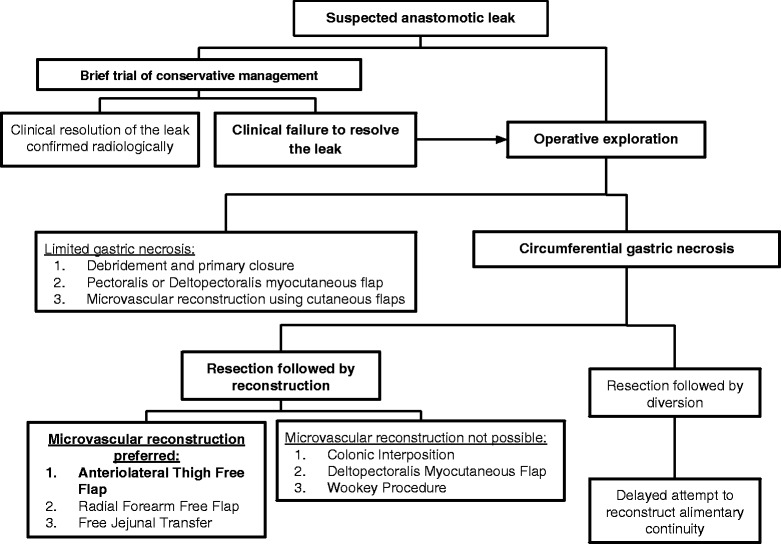
A decision tree for managing suspected anastomotic leaks following gastric pull up reconstruction after pharyngoesophagectomy. Decisions made in the case report are highlighted in bold

Based on the literature review and the presented case, a decision tree for managing suspected anastomotic leaks following GPU reconstruction of pharyngo-esophageal defects is presented (Fig. 4). This decision tree can also be used when considering rescue options for failed reconstructions other than GPU.

A high index of suspicion is required to recognize an anastomotic leak early. Signs that point to a potential anastomotic leak are edema, erythema, or tenderness of the neck skin flaps that present with a rising white blood cell count [23]. In the majority of cases, a suspected anastomotic leak can initially be managed conservatively with supportive care including nutritional support, antibiotic therapy, local wound packing and close observation [16]. Clinical judgment is required to decide on the length of conservative treatment, as prolonged exposure of neck structures or mediastinum to gastric secretions can lead to devastating consequences such as carotid blow out [24]. Once a trial of conservative treatment has failed, the patient has to be taken to the operating room for definitive management.

Prior to entering the operating room, it is helpful to consider various reconstructive options available for the patient. The reconstructive options will be dictated by the degree of anastomotic necrosis. The majority of anastomotic leaks result from limited areas of gastric necrosis and subsequent dehiscence [16]. After thorough debridement of devitalized tissue, most of the small defects can either be closed primarily or with local myocutaneous flaps [16].

A more challenging scenario is circumferential necrosis at the anastomotic site. In these situations, we advocate for the use of distant flaps and microvascular reconstruction. For some patients, however, microvascular reconstructive techniques are not possible. This could be due to a lack of healthy donor vessels, hemodynamic instability, or lack of available microvascular expertise. In these challenging scenarios, the options for reconstruction would include colonic interposition [15], deltopectoralis myocutaneous flap [25], Wookey procedure [26] or stoma diversion with delayed reconstruction [2].

If microvascular reconstruction is possible, the free tissue donor sites can be further divided as intra-abdominal versus extra-abdominal. The choice of the donor flap will depend on the length of the defect, the available vasculature, and the experience of the reconstructive surgeon. Intra-abdominal based free jejunal transfer are ideal for reconstructing long segments of esophagus as it provides peristalsis that later helps with swallowing [2]. However, in the setting of GPU rescue, we recommend against the use of intraabdominal flaps, which necessitate re-entery into a postoperative abdominal cavity. Other disadvantages include restricted trachea-esophageal voice and lower maximal dose of post operative radiation therapy [27, 28]. In the presented case, an extra-abdominal flap was selected as the patient had intra-abdominal adhesion, liver cirrhosis, and a remote colectomy. In the presented case, the ALT proved to be a robust flap for reestablishing alimentary continuity. The ALT flap has been shown to provide up to 40 cm of length for esophageal reconstruction, especially when folded in a conical fashion [2, 29]. Radial forearm free flap is an alternative for extra-abdominal free tissue transfer.

Any flow diagram or a decision tree is an over simplification of what is often a complex series of clinical decisions. Much depends on expert clinical judgment honed by years of clinical experience and availability of expertise in various reconstruction options. Nevertheless, as illustrated by the presented case, a general framework for making decisions serves as a helpful starting point in challenging cases.

## Conclusions

To the best of our knowledge, the presented case is the first ALT rescue of a failed GPU pharyngoesophageal reconstruction. The review of literature suggests that ALT reconstruction of the failed GPU should be one of the reconstructive options considered in the challenging cases of circumferential gastric necrosis.

## Abbreviations

ALT: Anterolateral Thigh Free Flap
GPU: Gastric Pull Up
